# Body Mass Index and 12-year Mortality Among Older Mexican Americans Aged 75 Years and Older

**DOI:** 10.1093/geroni/igaa057.3377

**Published:** 2020-12-16

**Authors:** Reshma Jadhav, Kyriakos S Markides, Soham Al Snih

**Affiliations:** The University of Texas Medical Branch at Galveston, Galveston, United States

## Abstract

The objective of this study was to examine the effect of body mass index (BMI) on 12-year mortality among older Mexican Americans aged 75 years and older. Data are from wave 5 (N=1,367) of the Hispanic Established Population for the Epidemiologic Study of the Elderly (2004/2005-2016). Measures included socio-demographics, self-reported medical conditions, body mass index (BMI), disability, Mini-Mental-State-Examination (MMSE), short physical performance battery (SPPB), high depressive symptoms, and falls. BMI was classified as underweight (< 18.5), normal weight (18.5 to < 25), overweight (25 to < 30), obesity type I (30 to <35), and morbid obesity (≥ 35).Cox proportional hazards regression analysis was performed to estimate the hazard ratio of 12-year mortality as a function of BMI categories at baseline. The average of the sample was 81.2 years, 17.2% were underweight, 22.7% were normal weight, 27.4% were overweight, 31.9% were obesity type I, and 38.6% were morbid obesity. Mexican Americans aged ≥75 years with overweight or obesity Type I had a reduced hazard ratio (HR) of death (HR=0.80, 95% CI=0.68-0.93 and HR=0.73, 95% CI=0.60-0.88, respectively) over 12-years of follow-up. The HR of death for underweight and morbid obesity participants was 1.78 (95% CI=1.13-2.80) and 1.03 (95% CI=0.79-1.36), respectively. Female participants and those with high scores in the MMSE and SPPB had decreased risk of death. This study confirmed the protective effect of overweight and obesity on mortality seen in this population at a younger age, which might have implications when treating older adults with overweight and obesity.

